# Patient guardians as an instrument for person centered care

**DOI:** 10.1186/1744-8603-10-33

**Published:** 2014-05-08

**Authors:** Lopa Basu, Ruben Frescas, Humphrey Kiwelu

**Affiliations:** 1 University of Chicago, Chicago, Illinois, USA; 2 Northwestern University McGaw Medical Center, Chicago, Illinois, USA; 3 Mbeya Referral Hospital, Mbeya, Tanzania

**Keywords:** Patient centered care, Patient guardian, Global partnerships, Reverse innovation, Advocacy, Health worker force, Patient education

## Abstract

Person-centered care involves keeping the person at the center of the care planning and decision-making process. While the theory behind person-centered care is commonly shared, its application in healthcare settings is more challenging. In a number of African countries, a lesson emerges involving the application of person-centered care through the use of patient guardians. Patient guardians, often family or close friends, act as an extension of the patient’s hospital care team. Medical teams engage with these self-designated individuals who invest their time and efforts in the care of the patient. More importantly, the guardian continues this role and relationship when the patient is released from the hospital to return home. Healthcare workers view patient guardians as a valuable resource. In a structured manner, guardians become stewards of information regarding topics such as hand hygiene and infection control. The knowledge gained can help the recovering patient upon discharge and potentially spread the information to others in the community. Further study of this model may show clear applicability to help improve health literacy in underserved settings in both low-income and high-income countries.

## Barefoot patient advocates

According to the Institute of Medicine, patient-centered care involves: “whole person” care; coordination and communication; patient support and empowerment; and ready access [1,2]. Further modification of this term includes a holistic concept defining the focus on the person, irrespective of their confines to the health facility in person centered care [3]. In low-income countries, access and patient empowerment are limited, posing unique challenges in promoting person centered care concepts. Our goal is to highlight a model focusing on educating patient guardians in an effort to enhance patient-support systems in the health facility and beyond. There is potential applicability of a patient guardian teaching model in high income countries whereby caretakers and legal guardians can be integrated into the patient care plan in a formal manner. As discussed by Syed et al. [4], there is limited understanding on developing to developed nation lessons. This patient guardian teaching model can foster learning from the direct experience of emerging economies.

During a recent evaluation of patient safety in Eastern Africa, a frequent finding was the utility of a patient guardian in the health facility setting. In the U.S., a patient guardian is usually defined as a legal decision maker for a patient unable to express oneself, or make health care decisions [5]. In African studies, guardians are commonly defined in the context of medication compliance in regards to direct-observation therapy (DOT) for TB and HIV treatment [6-8].

Currently in the literature, there is no established definition of a “patient guardian” specific to health facility care in low-income countries. In this context, a patient guardian is defined as either a patient’s relative, friend or less frequently a designated hospital-employee, operating as an extension of the patient’s hospital care support system and recognized as such by health care providers. Patient guardians in low-income countries are distinctly different from guardians in high-income countries as they are intimately involved in the patient’s care and often live on the hospital grounds due to distance and high transportation costs. In many African regions, patient guardians supplement an overburdened work force and are directly involved in the patient’s activities of daily living including bathing, feeding, cooking, transporting patients, and assisting with rehabilitation exercises. A guardian, integrated as extensions of the health care team, is seen as an asset by the health care staff. Similar to high-income countries, guardians are patient advocates answering questions from the medical team for those patients who are unconscious or unable to communicate.

Realizing the value of the patient guardian, medical teams in low-income health facilities have developed daily health education sessions, specifically for patient guardians. These sessions include topics such as hand washing and infection prevention. “The guardians play a critical role in the patient’s hospitalization and it is necessary for us to use this opportunity to educate them on basic infection prevention”, states a healthcare worker in Gondar University Hospital in Ethiopia. Many guardians live in remote areas and have limited health literacy, making them vulnerable to infections, such as cholera, when residing in the hospital patient guardian shelters. Hospital staff educate patient guardians daily during the patient’s hospitalization through structured teaching sessions thus promoting health literacy.

The role of patient guardians in an overburdened healthcare facility has been viewed as an asset in low-resource settings. In a report by USAID, most patients had preference for guardians to conduct direct observation therapy for medical treatment [9]. The study goes on further to recommend guardians to reduce workload on health staff and improve compliance [9]. Evidence also exists that guardians desire an active role in patient care. One report from Jhpiego found high marks from guardians themselves who were trained, being appreciative of the information given regarding infection prevention [10].

The emotional support a patient guardian provides for patients has also been viewed as an asset for the patient’s recovery process. Access to patients, whether as visitors or guardians, can decrease patient anxiety and improve patient satisfaction scores [11]. Although not a hospital, a Norwegian study done in nursing homes highlights the common vulnerability of patients with dementia who are often non-verbal and how patient relatives are a critical resource in an effort to strengthen the patient participation process [12].

The economic value of families’ contribution to the health system in the United States is widely acknowledged. According to a U.S. Congressional Budget Office report: “The value of donated care probably exceeds that of any other category of long term care financing but is difficult to quantify in dollar terms” [13]. Family members serve as a largely hidden extension of the workforce at the intersection of acute and long-term care delivery [14].

The development of health education models targeting patient guardians is an innovation that has emerged from a limited resource setting with great promise. Educating patient guardians, often family members, about infection prevention and encouraging the engagement of the patient support system is not just innovative, but common sense.

As attention towards a more integrated person centered care model grows, the development of a learning lab through a joint African and US partnership could foster further learning. Current literature is beginning to study how innovations such as global partnerships from low income country experiences may potentially transfer to high income countries [4].

The four basic principles to take away from the experience of patient guardians in the African context have been summarized in Table 1.

**Table 1:** The four basic principles to take away from the experience of patient guardians in the African context

Definition	A patient’s relative, friend or less frequently a designated hospital-employee, operating as an extension of the patient’s hospital care support system and recognized as such by health care providers
Value	Structured educational sessions empower guardians with healthcare knowledge that can be shared with their respective communities.
Education	The patient guardian teaching model occurs in the health facility, in a structured setting, and continues into practice in the community. This benefits the patient’s recovery at home, and helps set an example for others in their respective community.
Person Centered	Further study of this model may reinforce and support a person centered care and expanded chronic care model in the high-income countries [15].

The Alma Ata Declaration purposefully includes numerous health professionals and auxiliaries as part of the health team [16], and patient guardians should be included in these auxiliaries. The WHO health workforce group could assess the effects of patient guardian roles in overburdened health systems within low-income settings. As person centered care follows one outside the health facility, it is essential to learn models that emphasize the patient support system. A system that improves comfort, adherence and alleviates an overburdened health system needs inclusion of assets such as patient guardians to achieve the highest standard of care possible.

## Abbreviations

USAID: United States Agency for International Development; US: United States of America; WHO: World Health Organization; DOT: Direct Observation Therapy; HIV: Human Immunodeficiency Virus; TB: Tuberculosis.

## Competing interests

The authors declare that they have no competing interests.

## Authors' contributions

LB conceived the article and contributed to the writing; RF contributed to the writing; HK contributed to the writing. All authors read and approved the final manuscript.
